# Tobacco use and asking prices of used cars: prevalence, costs, and new opportunities for changing smoking behavior

**DOI:** 10.1186/1617-9625-4-2

**Published:** 2008-07-31

**Authors:** Georg E Matt, Romina Romero, Debbie S Ma, Penelope JE Quintana, Melbourne F Hovell, Michael Donohue, Karen Messer, Simon Salem, Mauricio Aguilar, Justin Boland, Jennifer Cullimore, Marissa Crane, Jonathan Junker, Peter Tassinario, Vera Timmermann, Kristen Wong, Dale Chatfield

**Affiliations:** 1 Department of Psychology, San Diego State University, San Diego, USA; 2 Joint Doctoral Program in Health Behavior Research, San Diego State University and University of California, San Diego, USA; 3 Graduate School of Public Health, San Diego State University, San Diego, USA; 4 Moores Cancer Center, University of California, San Diego, USA

## Abstract

Secondhand smoke (SHS) causes premature death and disease in children and adults, and the scientific evidence indicates that there is no risk-free level of exposure to SHS. Smoking tobacco in a car can pollute the microenvironment of the car with residual SHS, leaving telltale signs to potential buyers (e.g., odor, used ash tray). This study examined (a) the proportion of used cars sold in the private party market that may be polluted with tobacco smoke and (b) whether asking prices of smoker and nonsmoker cars differed for cars of otherwise equivalent value. A random sample of 1,642 private party sellers were interviewed by telephone, and content analyses of print advertisements were conducted. Findings indicate that 22% of used cars were advertised by smokers or had been smoked in during the previous year. Among nonsmokers, 94% did not allow smoking in their car during the past year. Only 33% of smokers had the same restrictions. The smoking status of the seller and tobacco use in the car were significantly (p < .01) associated with the asking price independent of a car's Kelley Blue Book value (KBB). Used nonsmoker cars were offered at a considerable premium above their KBB value (>11%) and above comparable smoker cars (7–9%). These findings suggest that community preferences are affecting the value of smoke-free cars. New directions for research, tobacco control policies, and health education are discussed to further reduce smoking behavior, to help consumers make informed purchasing decisions, and to protect nonsmokers from SHS exposure.

## Background

Secondhand smoke (SHS) contains more than 50 known human carcinogens and has recently been classified as toxic air contaminant [1,2]. SHS causes premature death and disease in children and adults, and the scientific evidence indicates that there is no risk-free level of exposure to SHS [1-3]. A growing number of local communities and states in the U.S. and in countries throughout the world are therefore adopting stricter policies to curb tobacco use in general and to reduce exposure to SHS exposure in particular [4-6].

The introduction of stricter tobacco control policies is often accompanied by health education campaigns about the harmful effects of tobacco use on smokers and vulnerable groups of nonsmokers [7-10]. The ultimate goals of these efforts are to improve public health through changing personal smoking behavior, community standards, and attitudes toward tobacco use and SHS [2,8,11,12]. According to the 2006 National Health Interview Survey [13], however, steady declines in smoking rates since the 1960s appear to have stalled, remaining unchanged at 21% since 2004. Thus tobacco control efforts remain a high public health priority, requiring renewed efforts to further reduce tobacco use and SHS exposure.

The present study examined the prevalence of tobacco use and asking prices of used cars in a community that has experienced extensive public health education campaigns since passing comprehensive statewide tobacco control legislation in 1988 [14]. We hypothesized that in such a community, smoke-free cars would be offered at a premium compared to smoker cars, controlling for other factors influencing the value of a car. If this is the case, future research may be warranted to better understand the effects of tobacco use on the value of personal property and how such consumer preferences could help further reduce tobacco use and SHS exposure.

### Tobacco Use in Cars

Compared to research on smoking restrictions in the workplace, restaurants, and at home, relatively little is known about smoking restrictions in cars. Existing research suggests that smoking restrictions in cars are less common than those at home. In California, two out of three family cars had a complete smoking ban in 1996 and 1998 [15,16], compared to almost four out of five homes with complete smoking bans. Among smokers, however, only 29% had a complete car smoking ban, and 43% had a complete home smoking ban. Similar patterns were observed in urban and rural settings of the U.S. outside of California. Halterman et al. [17] found that among urban households with smokers and children suffering from asthma only 64% had a complete ban on smoking in the home and 49% in the car. Kegler & Halinka Malcoe [18] examined low-income families of children in rural Oklahoma. They found that 49% of Native American households and 43% of Caucasian households banned smoking in the home, but only 35% and 40%, respectively, banned smoking in the car. A deviation from this pattern was reported by King et al. [19] among African-Americans who found a higher percentage of nonsmokers had car than home smoking bans (84% vs. 74%). Among smokers, however, only 17% and 21% had similar bans.

### Residual SHS Contamination of Used Cars

When tobacco is smoked in the confined environment of a car, tobacco smoke pollutants can reach extremely high levels [20]. Volatile SHS components absorb into surfaces within minutes of emission, contaminating objects with which they come in contact. Subsequently, this residual SHS (also known as aged SHS or third-hand smoke [21,22]) is re-emitted into the air over days, weeks, and months, accumulates in dust, and deposits on surfaces [23-27], creating a route of exposure for drivers and passengers of smoker cars in the absence of concurrent active smoking.

Unlike mechanical or electronic defects, detecting the signs of previous tobacco use in a car often requires little technical expertise from a potential buyer. Routine tobacco use leaves many telltale signs to prospective buyers. Foremost is a distinct odor caused by the re-emission of SHS contaminants from surfaces and dust that were polluted during active smoking. Matt et al. [28] have shown that SHS odor and ash marks are significantly associated with the residual contamination of dust, surfaces, and the air in cars. It is this odor and other visible signs that can signal to a potential buyer that a car has been smoked in and that can be difficult and expensive to remove through cleaning or repairs.

### Private Party Sales of Used Cars in the U.S

The used car market provides a particularly interesting opportunity to examine the value of a smoke-free personal environment, because a large and diverse cross-section of the general population in the U.S. sells and buys used cars. Moreover, a substantial portion of personal income is spent on the purchase and maintenance of cars. In 2005, 44.1 million used and 16.9 million new cars were sold in the U.S. [29,30]. These transactions totaled $780 billion, $367 billion of which were accounted for by used car sales. Approximately 30% of used cars were sold by private parties [30].

Although asking prices for used cars are often informed by published pricing guidelines (e.g., Kelley Blue Book), sellers can advertise their cars for any price, buyers can offer any price, and the eventual sales price is subject to the local market forces of supply and demand. Sellers typically begin gauging an asking price by establishing the standard value of their used car based on its make, model, age, mileage, and condition. In addition, sellers often look up the asking prices of similar cars currently offered for sale by consulting the classified ads of local newspapers and used cars offered by dealers. Sellers then apply additional adjustments for factors believed to affect the value of a car in the community where it is sold that were not – or not sufficiently – included in the standard model. These adjustments may increase (e.g., chrome wheels; smoke-free car) or decrease the value of a used car (e.g., no air conditioning, smoker car). Further price adjustments follow if a car fails to sell and the seller is unable to remediate problems preventing a sale.

The Behavioral Ecological Model (BEM) provides a theoretical framework of the association between the values and norms of communities and the behaviors and preferences of individuals. Briefly put, BEM postulates that culture-wide social contingencies influence health practices at both the individual and the community levels. Changes in norms (e.g., tobacco use in the presence of nonsmokers) can initiate a cascade of social contingencies from the population to the individual levels that affect the strength of a given cultural characteristic (e.g., tobacco tolerance) [31,32]. Consequently, changes in individual health behavior (e.g., car smoking ban) can be initiated by changing social and economic contingencies at the population (e.g., smoker cars are worth less in the private party market) and individual levels (e.g., family members complain about stale tobacco odor in car). This study offered an opportunity to explore hypotheses about emerging social contingencies with respect to tobacco. We reasoned that cultural changes regarding tobacco use should lead to lower prices for cars offered by smokers than equivalent cars offered by nonsmokers. This would create new social and economic contingencies affecting tobacco use and SHS exposure among seller, buyers, and passengers.

## Methods

### Participants

The target population was private party sellers (age ≥ 18 years) of used cars who advertised between January 2005 and April 2006 in the San Diego (USA) print editions of the Auto Trader magazine, a popular weekly publication of classified ads for used cars, and with phone numbers in the 619 and 858 area codes. Approximately 3,000 private party advertisements were published per week in the target area codes.

A random sample of 100 pages was drawn each week across all Auto Trader issues for different types of automobiles (i.e., domestic, Asian, European, sport utility models, newer and older models of trucks and vans) using a random number generator. The selected pages were sorted in the order in which the random numbers were generated. We called all eligible sellers on a page in the order of the sorted pages until we had recruited the target number of smokers each week (1, 2, or 3). Of the 2,590 sellers who were screened by phone for a study about the SHS contamination of cars, 2,081 (80%) reported their smoking status, 1,667 (64%) reported the smoking status of the car, and 1,642 (63%) reported both. For analyses of asking prices (N = 1,425), we excluded cars built before 1989 because car values could not be determined reliably. Table 1 provides information about asking price differences in the print advertisements, mileage, age, and make of cars by smoking status of the car and the seller. The Institutional Review Board at San Diego State University approved the research protocol.

**Table 1:** Asking price, Kelley Blue Book value, mileage, age, and make of used cars, and percentage of used cars sold by a smoker and cars in which cigarettes have been smoked.

	All	Smoke-Free Car		Smoker Car	
		
		Nonsmoker Seller	Smoker Seller	Nonsmoker Seller	Smoker Seller
Sample size	1,642	1,274	95	77	196
% of Used Cars		77.6	5.8	4.7	11.9
Asking Price^a ^($)	7,636	8,114	7,684	5,684	5,602
KBB Value^a,b ^($)	6,906	7,213	7,188	5,330	5,734
Mileage^a^	69,339	68,019	72,099	87,400	70,709
Age^a ^(Years)	6.0	5.9	6.0	6.0	6.7
Make					
% American	50	51	40	48	48
% Japanese	33	32	39	43	34
% European	17	17	20	9	18

^a ^geometric means

^b ^KBB: standard value of used car for private party sales based on Kelley Blue Book

### Measures

#### Telephone interview

Sellers were identified as smokers if they reported smoking cigarettes every day or on some days during the past year. Cars were identified as smoker cars if the seller reported that one or more cigarettes had been smoked in the car during the past year.

#### Content analysis of print advertisements

Data about the asking price, year, make, model, mileage, condition, and special features were obtained from the printed advertisement of the car or during the phone interview. For each car, the widely used KBB value was determined given the information provided in the printed advertisement, using the online valuation calculator [33]. If mileage information was omitted (15% of cars), the KBB value was determined assuming 15,000 miles per year. While year, make, and mileage can be easily determined, condition, appearance, and special features often required judgment and interpretation. The condition of a car was coded as "good", unless the seller listed specific negative or positive characteristics, in which case the condition was downgraded to "fair" or "poor" or upgraded to "excellent". No KBB values are available for cars in "poor" condition (e.g., salvage title, major mechanical problems). When this was the case, a car was excluded from analyses (<1%). To examine the reliability of the KBB value determination by coders of this study, intraclass correlations were calculated based on a random subsample of 50 cars coded by each of five coders. The ICC for individual ratings was 0.93, indicating that KBB values were determined with good reliability.

### Statistical Analyses

Statistical analyses were conducted using Stata version 9.2 [34]. Asking price and KBB values were log-transformed to normalize the model residuals. This was confirmed through graphical and quantitative analyses. Because cars sold by smokers and nonsmokers may differ in characteristics other than smoking status, it was important to adjust for these factors. Multiple regression analyses were conducted, in which the log-transformed asking price was the response variable and the log-transformed mean-centered KBB value and its quadratic and cubic terms were entered as covariates to statistically control for differences in the asking prices given the KBB valuation. We then investigated whether sellers may have applied different weights to the components of the standard valuation. Thus we added mileage, year, make, and condition of a car as additional covariates, and retained covariates in the model *α *< 0.05. Finally, we entered dummy variables for smoking status of the seller, smoking status of the car, and interaction terms of all covariates and explanatory variables. No interaction effects were statistically significant (*α *= 0.05). We examined the robustness of model estimates through sensitivity analyses in which different transformations of asking prices and KBB values, robust variance estimates [35], bootstrapped regression coefficients, and alternative regression models (quantile and robust regression) were explored. Throughout these analyses, overall model fit, statistical significance for smoking status of sellers and cars, and effect sizes remained stable. The reported findings are based on log-transformed variables and models specifications outlined above.

We derived maximum likelihood estimates based on our sampling design to estimate the proportions of smokers and smoker cars in the target population. Briefly, we modeled the number of smokers who were not recruited and the number of smokers who were recruited using the negative binomial distribution. Variance estimates were derived via the delta method [36].

## Results

### Smoking Status of Sellers and Cars

Overall, 17.7% (95% Confidence Interval: 15.9; 19.6) of sellers reported themselves being smokers, 16.6% (14.8; 18.4) of used cars had reportedly been smoked in, and 22.4% (20.4; 24.4) of cars had either been smoked in or were being sold by a smoker. Among the nonsmokers, 5.7% (4.6; 7.1) had allowed smoking in their car during the previous year. Among smokers, 67.4% (61.6; 72.6) had allowed smoking in their car. Table 1 provides additional detail on the smoking status of sellers and their cars.

### Smoking Behavior and Asking Price

Linear regression models showed that the KBB value accounted for 84% of the variance in asking price (*p *< 0.001), indicating that the asking prices closely matched the prices suggested by the KBB valuation model. In addition, there were statistically significant quadratic and cubic trends (*p *< .001; accounting for additional 1% variance). Further investigations of the nonlinear associations indicated that for cars with low KBB values (<$2,500) sellers raised the asking price comparatively more than for more expensive cars. Sellers made another adjustment based on the make of a car that went beyond the standard KBB valuation model, accounting for an additional 1% of the variance in asking price (*p *< 0.001).

Controlling for KBB value and make of car, smoking status of the seller and the car accounted for a significant proportion of variance (*F*(2, 1388) = 6.37; *p *= 0.002). Because the two variables were highly correlated (*r *= 0.62; *p *< .001), neither accounted for variance independent of the other when entered jointly in the model. When examined in separate models, smoking status of the car (*t*(1391) = 3.26; *p *= 0.001) and of the seller (*t*(1389) = 3.09; *p *= 0.002) each accounted for significant proportions of variance in asking price (approximately 0.1%) independent of KBB value and make of car. We address the practical significance of this effect below.

**Figure 1:**
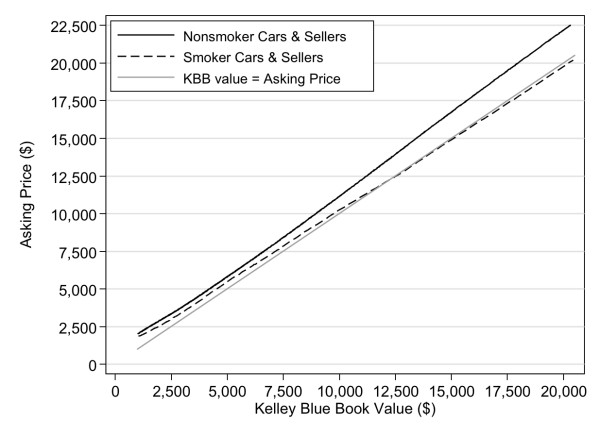
**Association between asking price and Kelley Blue Book value of used cars sold by smokers in which cigarettes have been smoked and by nonsmokers in which no cigarettes have been smoked (LOWESS fit lines)**. Also reported is a reference line for cars offered at the KBB value.

Figure 1 shows the association between KBB value and asking price for cars sold by smokers in which tobacco had been smoked and cars sold by nonsmokers in which no tobacco was smoked. Also shown is the reference line indicating cars offered at their standard KBB value (i.e., asking price = KBB value). The figure shows that the asking prices of smoker cars were consistently lower than those of nonsmoker cars of equivalent KBB value and were on average within ± 4–5% of their standard KBB value. In contrast, nonsmoker cars were consistently offered at a 10–13% premium above their KBB value.

Table 2 provides model estimates of asking prices based on smoking status of car and seller for cars at different KBB values, controlling for make, model, age, mileage, and condition of car. These estimates reveal that cars sold by smokers and cars that had been smoked in were offered at a significantly lower price than equivalent cars offered by nonsmokers and in which no smoking was reported. Because the regression models used log-transformed asking prices, the observed effects of smoking status of a car and a seller can be interpreted as differences in the percentage of asking price. That is, given a particular KBB value and model of car, the value of a car decreased by 7.7% if it had been smoked in compared to a car that was smoke-free. The value decreased by 7.5% if a car was sold by a smoker compared to an equivalent car sold by a nonsmoker. Finally, a car decreased in value by 9.0% if it was sold by a smoker who allowed smoking in the car compared to an equivalent car sold by a nonsmoker who prohibited smoking.

**Table 2:** Adjusted asking prices for cars of smokers and nonsmokers at different KBB values.

KBB Value^a^		Adjusted Asking Price in U.S. $^b^			
		
Decile	U.S. $	Nonsmoker Car & Seller	Smoker Seller	Smoker Car	Smoker Car & Seller
1	2,080	2,893	2,677	2,670	2,634
2	3,223	4,031	3,731	3,720	3,670
3	4,354	5,187	4,803	4,787	4,722
4	5,674	6,563	6,081	6,058	5,976
5	7,363	8358	7,747	7,715	7,609
6	9,186	10,324	9,574	9,531	9,399
7	11,818	13,200	12,247	12,189	12,018
8	14,900	16,600	15,409	15,333	15,113
9	20,515	22,823	21,200	21,093	20,779
% Difference: Asking Price vs. Nonsmoker Car & Seller		Referent	-7.0%	-7.7%	-9.0%
% Difference: Asking Price vs. KBB at median value ($7,363)		+13.5%	+5.2%	+4.8%	+3.3%

^a^Kelley Blue Book (KBB) values in U.S. currency for 1^st ^to 9^th ^deciles.

^b^Asking prices were adjusted for linear, quadratic, and cubic effects of KBB values and make of car.

Table 2 also shows the percentage premium over KBB value that sellers asked for. For a car of median KBB value (i.e., $7,363), nonsmokers who had not smoked in their car asked for a 13.5% premium over the KBB value. This compared to a 3.3% premium for an equivalent car sold by a smoker whose car had been smoked in.

## Discussion

To the best of our knowledge this is the first study to examine the association between tobacco use in cars and their asking price in the private party used car market. Our findings show that one out of five used cars for sale in the San Diego (CA) metropolitan area were offered by smokers or had been smoked in during the previous year. While nine out of ten nonsmokers reported that no cigarettes had been smoked in their car during the past year, only one in three smokers reported to have had such a restriction. Finally, used nonsmoker cars were offered at a considerable premium above their KBB value and above comparable smoker cars. In the following we will briefly discuss limitations of this study and implications for tobacco control and consumer protection in the used car market.

### Limitations

Findings from this study are based on a cross-sectional survey and self-reported information. Because of the social undesirability of smoking behavior and its likely negative impact on the sale of a car, we suspect that some misreporting took place such that the proportion of smoking sellers and smoker cars are likely to be higher than we determined.

While plausible, a preference for smoke-free personal environments is not the only possible explanation for the observed differences in asking price. The non-experimental nature of this study raises the possibility that the observed differences may be due to additional variables that affect both smoking behavior and the value of the car. To address this issue we included in our analyses as a covariate the standard KBB value of a vehicle. It is possible, however, that smoker cars overall are in worse condition than nonsmoker cars and are so in a way that was not measured by our rating of the condition and appearance of a car based on its print advertisement. It is also possible that potential buyers use the smoking-status of a seller or the tobacco odor of the car as a proxy of poor maintenance to negotiate a lower price and are not at all concerned about potential health effects or odor nuisance. Our study was also not able to examine whether smoking and nonsmoking buyers have similar preferences and how smoking status of the seller and tobacco odor of the car are considered in the purchasing decision.

This study focused on asking price and does not allow conclusions about actual sales prices. Although we expect that the sales price differences will exceed asking price differences, this should be demonstrated in future research. This study cannot provide explanations for the processes leading to the observed asking price differences. We also cannot rule out that smokers differed from nonsmokers in how they discounted asking prices for damages other than smoking or special features of their cars.

### Tobacco Use in Private Cars

Our findings support existing research that smoking restrictions for private cars are less common than for homes. In 2005, 53% of California's smokers reported living in a smoke-free home[37], but only 32% of the smokers in the present study reported that no cigarettes were smoked in the their car during the previous year. The relatively low prevalence of smoking bans in the cars of smokers raises the question whether cars may have become sanctuaries for smokers to light a cigarette while driving to and from places with smoking restrictions. While this smoking pattern protects nonsmokers when smokers drive alone, recent research [28] has demonstrated that it leads to the pollution of cars with residual SHS long after cigarettes have been smoked. Future research is needed to examine smoking behavior and restrictions in cars and how they may be influenced by restrictions in a smoker's home, workplace, and community.

### Tobacco Use and the Resale Value of Cars

Our findings indicate that smoker cars have lower asking prices than comparable nonsmokers cars. From the perspective of the BEM, such asking price differences are to be expected in communities that value smoke-free personal environments, creating a greater demand for smoke-free cars such that sellers can ask for a premium over the car's KBB value and over comparable smoker cars. In contrast, asking price differences would not be expected in communities tolerant of or indifferent to SHS exposure and tobacco odor in cars and their effects on health and the value of a car. Consistent with the BEM, asking price differences contribute to a community-wide culture that encourages car smoking bans and discourages overall tobacco use. Thus, even if the motivation for discounting asking prices is not a perceived health risk, the financial consequences may contribute to establishing community-wide norms for not smoking in cars. This adds one more setting in which smoking may become socially unacceptable.

This study was conducted in a community in Southern California that has been highly sensitized to the health effects of tobacco use in general and SHS exposure in particular over 20 years of public health education efforts [38]. These efforts have contributed to reducing smoking prevalence in California from 26% to 14% between 1984 and 2005 [37], and, in 2002, 93% of nonsmokers and 83% of smokers agreed that any exposure to SHS can be harmful to your health [12,39]. Our findings support the hypothesis that changes in collective values, smoking behavior, and attitudes toward SHS have influenced the market place, affecting the value of personal property and shaping purchasing decisions. Thus, monetary value of smoke-free environments in the market place may provide a useful outcome to evaluate long-term effects of tobacco control efforts at the level of communities. Because the health outcome of exposure to residual SHS in a car are not well understood, the observed differences in asking price may be a sign that the concerns of nonsmokers reach beyond recognized health risks and include concerns about the depreciation of personal property and quality of life. This is consistent with the increasing public debate about drifting smoke in multi-unit housing [40] and recent changes in local ordinances that led to smoking bans in city parks, on playgrounds, beaches, and sidewalks.

### Future Tobacco Control Efforts and the Used Car Market

From the perspective of tobacco control policies, the observed asking price differences are not only important outcomes of successful health promotion campaigns, but may suggest new strategies to further reduce tobacco use and SHS exposure. For instance, future tobacco control efforts could educate consumers about the effects of tobacco use on the value of used cars. Our findings suggest that many sellers are already cognizant of this effect. However, it is unclear whether this is equally known among smokers and nonsmokers and across different education, socio-economic, and ethnic groups. Health education campaigns could help motivate smokers to smoke less or quit altogether. Such campaigns could also empower consumers to assert their interests in smoke-free environments and in obtaining an appropriate discount if they choose to tolerate a smoker environment. Finally, consumer education campaigns would provide incentives to private sellers and dealers to advertise the smoking status of cars, allowing consumers to make informed purchasing decisions.

A more drastic approach would involve a change in the valuation model used by private parties, car dealers, banks, and insurance companies to value cars. Although it could be argued that such a step is unnecessary given the existing market response, it is worthwhile to consider this potential path as an explicit recognition of how a community values a car that may affect the health and driving experience of drivers and passengers.

Signs of tobacco use (e.g., odor, burn marks) are currently implicitly included among many factors that diminish the value of a car via their impact on appearance and overall condition. Our findings suggest that the smoking history of a car affects its value as much as many prominent features of a car that the KBB valuation models does consider. In November 2006, for instance, a 2000 Toyota Camry LE four-door sedan, 4-cylinder engine, automatic transmission, 77,000 miles, standard equipment, and in good condition was valued for private-party sale in the San Diego market at $7,695. If this car had been offered by a smoker and had been smoked in, the asking price would have been about $700 lower (i.e., 9%). For this car to loose $700 in KBB value, the car would have to miss all of the following standard features: air conditioning, power steering, power windows, power door locks, cruise control, and the dual front airbags. Admittedly, a car missing all of these features would probably sell in the market place for a much larger discount. Still, ignoring tobacco use in the valuation model of used cars disregards a feature of an automobile to which at least some communities appear to have assigned a considerable monetary value. From the perspective of the BEM, such a recognition would introduce an explicit incentive that may trigger further changes on the community and individual levels to reduce tobacco use and SHS exposure.

## Authors' contributions

GEM conceived and designed the study, performed the statistical analyses, and drafted the manuscript. RR and DSM contributed to the design and coordinated the study, participated in the data collection and data analysis. PJEQ and MFH contributed the design of the study and drafted the manuscript. MD and KM contributed to the data analysis and drafted the manuscript. SS, MA, JB, JC, MC, JJ, PT, VT, and KW contributed to the design the study and participated in the data collection. DC contributed to the design of the study. All authors read and approved the final manuscript.
